# Uncovering specific taxonomic and functional alteration of gut microbiota in chronic kidney disease through 16S rRNA data

**DOI:** 10.3389/fcimb.2024.1363276

**Published:** 2024-04-19

**Authors:** Yangyang Zhang, Weicong Zhong, Wenting Liu, Xiaohua Wang, Gan Lin, Jiawen Lin, Junxuan Fang, Xiangyu Mou, Shan Jiang, Jiayuan Huang, Wenjing Zhao, Zhihua Zheng

**Affiliations:** ^1^ Department of Nephrology, Center of Kidney and Urology, The Seventh Affiliated Hospital, Sun Yat-Sen University, Shenzhen, China; ^2^ Shenzhen Key Laboratory for Systems Medicine in Inflammatory Diseases, School of Medicine, Shenzhen Campus of Sun Yat-Sen University, Shenzhen, China

**Keywords:** chronic kidney disease, gut microbiota, 16S rRNA, biomarker, probiotics

## Abstract

**Introduction:**

Chronic kidney disease (CKD) is worldwide healthcare burden with growing incidence and death rate. Emerging evidence demonstrated the compositional and functional differences of gut microbiota in patients with CKD. As such, gut microbial features can be developed as diagnostic biomarkers and potential therapeutic target for CKD.

**Methods:**

To eliminate the outcome bias arising from factors such as geographical distribution, sequencing platform, and data analysis techniques, we conducted a comprehensive analysis of the microbial differences between patients with CKD and healthy individuals based on multiple samples worldwide. A total of 980 samples from six references across three nations were incorporated from the PubMed, Web of Science, and GMrepo databases. The obtained 16S rRNA microbiome data were subjected to DADA2 processing, QIIME2 and PICRUSt2 analyses.

**Results:**

The gut microbiota of patients with CKD differs significantly from that of healthy controls (HC), with a substantial decrease in the microbial diversity among the CKD group. Moreover, a significantly reduced abundance of bacteria Faecalibacterium prausnitzii (F. prausnitzii) was detected in the CKD group through linear discriminant analysis effect size (LEfSe) analysis, which may be associated with the alleviating effects against CKD. Notably, we identified CKD-depleted F. prausnitzii demonstrated a significant negative correlation with three pathways based on predictive functional analysis, suggesting its potential role in regulating systemic acidbase disturbance and pro-oxidant metabolism.

**Discussion:**

Our findings demonstrated notable alterations of gut microbiota in CKD patients. Specific gut-beneficial microbiota, especially F. prausnitzii, may be developed as a preventive and therapeutic tool for CKD clinical management.

## Introduction

Chronic kidney disease (CKD) is a worldwide healthcare burden featured by lasting abnormalities in renal function or structure for at least 3 months, often due to primary, secondary, or hereditary kidney diseases (Webster et al., 2017; Kalantar-Zadeh et al., 2021). In recent years, the global prevalence of CKD has been steadily increased, affecting approximately 13.4% of the world’s population (Lv and LX, 2019). The high incidence has led to significant medical and economic burdens (Liyanage et al., 2022). CKD typically develops slowly without noticeable symptoms in its early stages. However, as the disease progresses, life-sustaining treatments such as dialysis and kidney transplantation become necessary (Levey et al., 2009). Early detection and effective early interventions of CKD patients can minimize the risk of kidney failure and major cardiovascular events (James et al., 2010). Therefore, there is an urgent need to develop novel biomarkers and innovative strategies that facilitate early detection and prevention of CKD.

The potential role of the gut microbiome is pivotal in maintaining human physiological homeostasis and health (Clemente et al., 2012). The emergence of notions such as “intestinal-renal syndrome” (Ritz, 2011) and “gut-kidney axis” (Meijers and Evenepoel, 2011) have provided new avenues for investigating the relationship between gut microbiota and kidney disease. Recent research has demonstrated that a significant alteration in the composition of gut microbes in CKD patients (Vaziri et al., 2013; Wu et al., 2021), indicating that the gut microbiota can serve as innovative targets for interventions. Research findings have indicated that patients with end-stage renal disease (ESRD) exhibit a significant reduction in the overall microbial quantity, and these alterations have been linked to the inflammatory state of the disease and renal function (Jiang et al., 2017; Li et al., 2019). Indoxylsulfate (IS), p-cresylsulfate (pCS) and phenylacetylglutamine (PAG) can serve as early indicators of renal dysfunction, with detectable changes in the gut microbiota associated with these metabolites in early kidney disease (Remuzzi et al., 2015). These findings provide promising insights into potential strategies for kidney disease management through the manipulation of the gut microbiome.

Several factors during CKD, such as inadequate elimination of uremic toxins, medications and antibiotics use, and dietary restrictions, can cause a uremic environment that can further impact the gut microbiota and disrupt the integrity of the intestinal epithelial barrier (Vaziri et al., 2013; Hobby et al., 2019; Steenbeke et al., 2021; Noce et al., 2022). Furthermore, the gut microbiota itself accelerates the deterioration of CKD by producing precursor toxins via the fermentation of dietary aromatic amino acids (Gryp et al., 2020). Taking these findings together, it appears that gut microbiota and kidney function are bidirectional. Even so, functional alterations in gut microbiota and their interaction with metabolic changes associated with CKD remain unclear. Ren et al. found that *Blautia* decreased in CKD (Ren et al., 2020), while another study found this genus increased in patient cohort (Liu et al., 2021). The particular alterations in the microbial community of CKD have long been a subject of controversy. Factors such as study projects, geographic location, sequencing platform, sequencing region, and analytical method influenced the intergroup differences. In comparison to individual studies, datasets from multiple studies are capable of comprehensively detecting alterations in the composition of microbiota related to CKD through the augmentation of sample size and the elimination of confounding factors. Therefore, conducting integrative analysis on multicenter across different nations with standardized methods can help address these research questions. In this study, we systematically integrated studies concerning the gut microbiota of CKD patients and collected existing 16S rRNA sequencing data from PubMed, NCBI and GMrepo databases. Through rigorous bioinformatics analysis, we examined the variations in bacterial diversity, relative abundance of microbial taxa, and the major metabolic functions within the gut microbiota of CKD patients compared to HC. The results of this study are important for the identification of microbial taxa that may contribute to the development of CKD and serve as future targets that can be targeted for prevention and treatment.

## Materials and methods

### Study search, selection, and inclusion

We performed thorough literature searches using various databases including PubMed (https://pubmed.ncbi.nlm.nih.gov), Web of Science (https://www.web of science. com/wos), and GMrepo (https://gmrepo.humangut.info), employing a combination of MeSH terms and free words. The GMrepo database (Wu et al., 2020) houses meticulously curated and consistently annotated human gut metagenomes, providing easily accessible and highly reusable data. The search strategy and search strings are presented in **Supplementary Table A1**. Our study considered publications available in the database from its inception up to January 1, 2023, with no language restrictions imposed during the search process. The inclusion and exclusion criteria of this study are shown in **Supplementary Table A2**.

### Datasets collection and bioinformatics analysis

**Figure 1:**
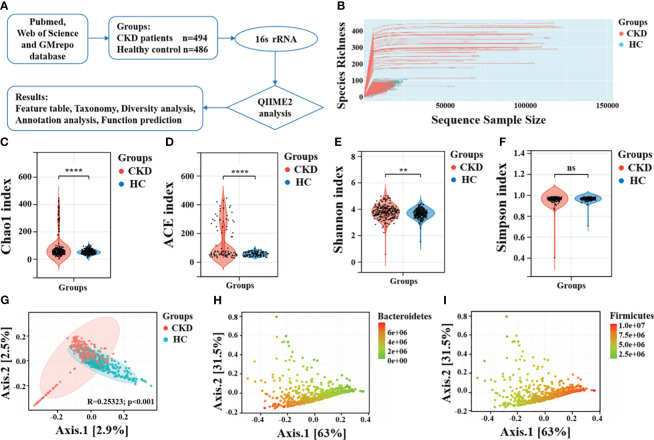
Taxonomic diversity profiling of gut microbiota. **(A)** Overview of the workflow based on 16S rRNA microbiome data. **(B)** The relationship between the number of samples and the number of species is shown by the rarefaction curve. Alpha diversity comparison between CKD and HC using the **(C)** Chao1 index, **(D)** ACE indices, **(E)** Shannon index and **(F)** Simpson index. ****, *P* < 0.0001; **, *P* < 0.01; ns, *P* > 0.05. **(G)** A PCoA plot using the Bray-Curtis dissimilarity metric. The same PCoA plot with color gradients according to the abundance levels of **(H)** phylum Bacteroidetes and **(I)** phylum Firmicutes.

The raw sequence data and metadata were obtained from the GMrepo database, European Nucleotide Archive (ENA), and NCBI Short Read Archive (SRA). Relevant literature was examined to collect metadata, and the samples were divided into two groups, CKD and HC, for further comparison. QIIME2 (Bolyen et al., 2019) was utilized to perform the bioinformatics analysis of the gut microbiota, involving the processing of all reads of the 16S rRNA gene sequences. Briefly, we first imported the raw data FASTQ files into a format compatible with subsequent processing in QIIME2. DADA2 algorithm (Callahan et al., 2016) was utilized for quality control, trimming, denoising, and assembly of the raw sequences to remove phiX, chimeric, and erroneous reads. Features were filtered based on ≥ 4 counts and 20% prevalence in samples, and low variance based on 10% inter-quantile ranges. When the lower bound of CI_99%_ of a unique sequence falls below zero, the sequence is considered unreliable, and then subjected to abundance filtering. Subsequently, we scaled data using total sum scaling to perform data normalization. Denoising and amplicon sequence variant (ASV) generation were completed using the parameters (trim-size = 435; min-size = 1; max-size = 460). Then, the representative sequences of the ASVs were aligned to the pre-trained GREENGENES database of version 13_8 with 99% similarity using the QIIME2 feature-classifier plugin, generating a taxonomic classification information table for the species. **Figure 1A** shows the workflow of this study.

### Microbial community taxonomic and functional profiling

Before performing downstream diversity analysis and comparing the relative abundances of taxonomic groups, a rarefaction depth cutoff of 400 was selected. A rarefaction curve analysis was performed to vividly evaluate the sequencing depth in relation to the number of operational taxonomic units identified. Alpha diversity was assessed using various indices including Chao1, ACE, Shannon, and Simpson diversity. Beta diversity was visualized through PCoA using Bray-Curtis dissimilarity. Permutational multivariate analysis of variance was examined for significant microbiomic differences between the two groups. The LEfSe method, which uses the non-parametric Kruskal-Wallis test, was employed to identify features that were significantly different between various groups. A LDA score (log10) of 2.0 was selected as the cutoff to indicate significantly enriched taxa. It was assessed whether a combined microbial biomarker could effectively differentiate between CKD and control groups using random forest models, which evaluates the significance of every feature by measuring the increase in classification errors when permuted (Liu et al., 2023). Furthermore, The ROC curve is used to evaluate the performance of a classifier by plotting the curve of true positive rate (sensitivity) and false positive rate (1-specificity) varying with the threshold and calculating metrics such as the area under the curve (AUC). We employed 100-fold cross-validation to reduce randomness and assess the accuracy of the random forest model in distinguishing between CKD and HC. Lastly, As a tool for functional annotation, PICRUSt2 was used for KEGG pathway analysis of gut microbiota. For the statistical analysis, *p* value significance < 0.05 was considered as a criterion.

## Results

### Literature investigation and inclusion

**Figure 2:**
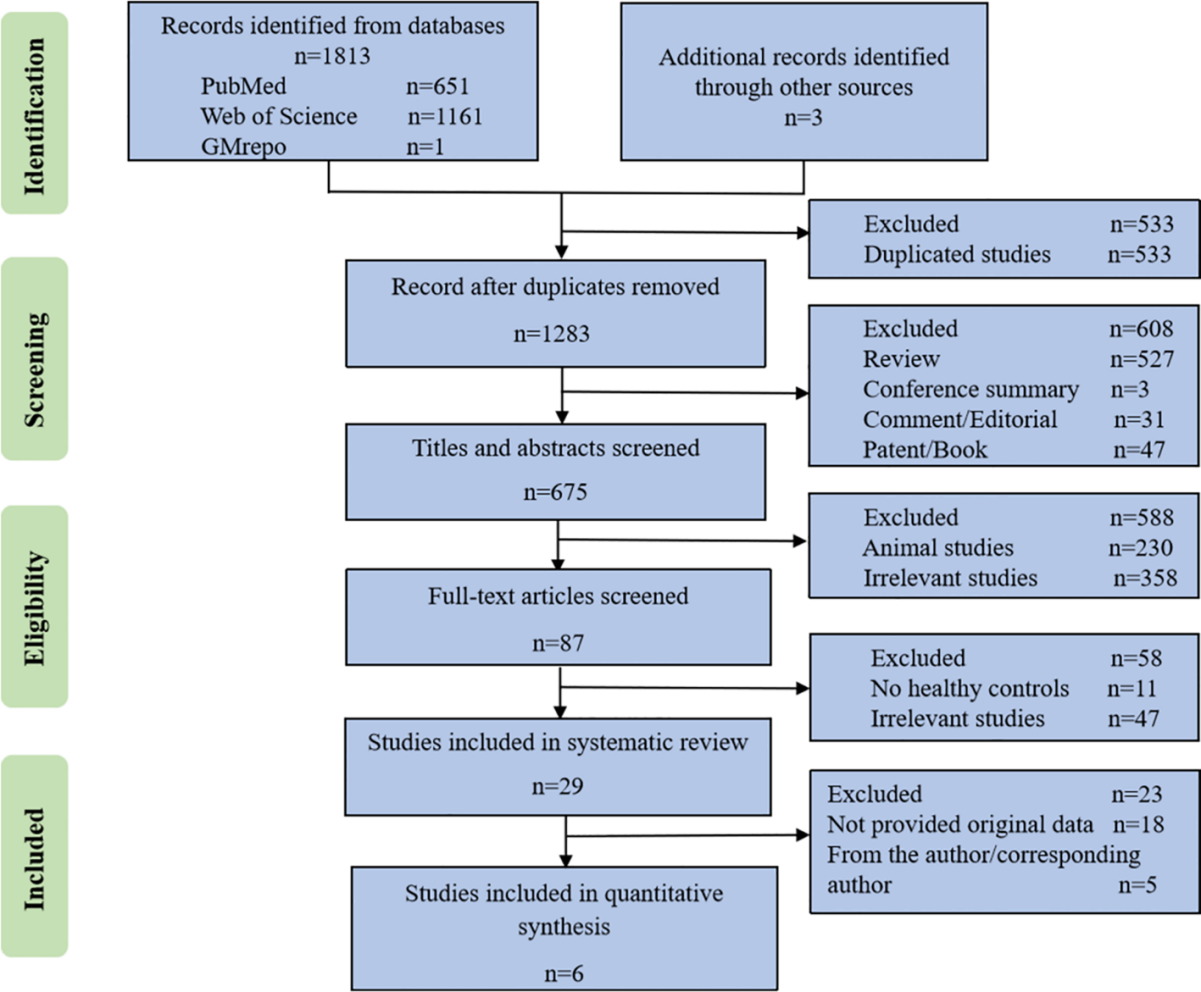
The flowchart of study selection.

The literature searches yielded 651 records from PubMed, 1161 records from Web of Science, 1 project from GMrepo and 3 additional records identified through other sources. After eliminating duplicate studies, a total of 1283 studies were identified. Following a thorough full-text review, 87 studies were selected for detailed analysis, out of which 29 studies met the requirements for qualitative analysis. The flowchart detailing the study selection is presented in **Figure 2**. Six microbiota studies labeled as S1 to S6 (Stadlbauer et al., 2017; Margiotta et al., 2020; Li et al., 2022; Zhou et al., 2022), all of which included 16S rRNA gene sequencing data, fulfilled the criteria for further quantitative analysis.

### General characteristics and biochemical indexes of the included datasets

**Table 1** and **Supplementary Table A3** demonstrated the general characteristics and biochemical indexes of the study populations in the included datasets. The combined data sets encompassed a total of 980 participants, consisting of 494 individuals with CKD and 486 healthy people. The sample sizes of the studies analyzed in this paper varied from 20 to 526 participants. Illumina sequencing platforms were employed across all studies. The sequencing regions encompassed V1-V2, V3-V4, and V4-V6. Countries where these studies conducted were China, Austria, and Italy. Except for the study S4, controls were comparable in age, gender, and BMI to both groups.

**Table 1:** Characteristics of included projects in the quantitative synthesis.

ID	Study	Numbers	Analysis methods	Sample storage	DNA extraction	PCR primers	Variable regions	Platform	Data analysis	Country	Data availability
S1	Liu et al., 2021.	237	16S rRNA	−80°C	DNeasy PowerSoil Pro Kit (QIAGEN, Germany)	338F/806R	V3-V4	NovaSeq PE250 platform	VSEARCH	China	SRA: SRP279052
S2	Ren et al., 2020.	489	16S rRNA	−80°C	Quick gel extraction kit (QIAGEN, Germany)	338F/806R	V4-V6	Illumina MiSeq platform	USEARCH	China	ENA: PRJNA562327
S3	Stadlbauer et al., 2017.	51	16S rRNA	−80°C	MagNA Pure LC DNA Isolation Kit III(bacteria, fungi)	27F/357R	V1-V2	European Nucleotide Archive	QIIME	Austria	SRA: PRJNA390475
S4	Margiotta et al., 2020.	78	16S rRNA	-80°C	FastDNA™ SPIN Kit for Soil (MP Biomedicals, Switzerland)	343F/802R	V3-V4	Illumina MiSeq platform	QIIME	Italy	ENA: PRJEB35666
S5	Li et al., 2022.	105	16S rRNA	NA	NA	NA	NA	Illumina NovaSeq 6000	QIIME	China	ENA: PRJNA797660
S6	Zhou et al., 2022.	20	16S rRNA	−80°C	E.Z.N.A. ^®^Stool DNA Kit (D4015, Omega, Inc., USA)	341F/805R	V3-V4	Illumina NovaSeq platform	QIIME	China	ENA: PRJNA772031

SRA, sequence read archive; ENA, European nucleotide archive. V1, V2, V3, V4, V5,V6, variable regions of the 16S rRNA gene; NA, Not available.

### Taxonomic diversity profiling of gut microbiota

The rarefaction curve showed the curve initially rose rapidly and then tended to levels off, indicating that sequencing depth we achieved captured a substantial amount of information on total species richness (**Figure 1B**). The results of alpha diversity revealed significant statistical differences in Chao1 index (*P* < 0.001), ACE index (*P* < 0.001), and Shannon diversity index (*P* < 0.01), indicating notable differences in both richness and evenness of the gut microbiota between CKD and HC (**Figures 1C-E**). Interestingly, there was no significant difference in the Simpson diversity index between the two groups (*P* = 0.44, **Figure 1F**). These findings aligns with those reported by Ren et al. (Ren et al., 2020) and Liu et al (Liu et al., 2021). The significantly decreased gut microbial richness and evenness may be may be linked to progressive renal injury, and the decline in microbial diversity among CKD patients could be attributed to the predominant use of antibiotics. The gut microbiota of CKD patients, which is typically characterized by a more evenly distributed, complex, and coordinated microbial community, becomes more singular and dominated by specific bacterial populations. Principal coordinate analysis (PCoA) plot revealed that the gut microbiota composition had significant differences between individuals with CKD and HC (**Figure 1G**). Moreover, the overall differences between the two groups is related to abundances of phylum Bacteroidetes and phylum Firmicutes (**Figures 1H, I**).

### Taxonomic annotation analysis of gut microbiota

**Figure 3:**
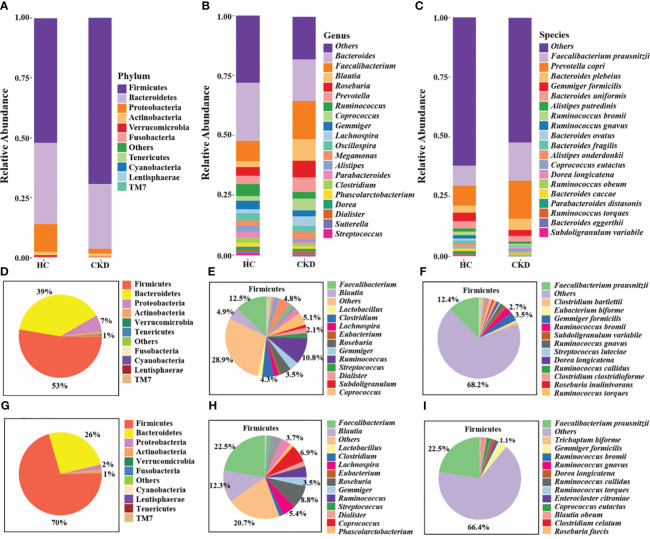
Annotation analysis of gut microbiota species. Compositional bar plot for the relative abundance of microbiota at **(A)** phylum, **(B)** genera, and **(C)** species levels across samples (the top 20 in relative abundance). Interactive pie chart for the proportions of taxonomic composition of the samples shown on the left **(D, G)** and the proportions of the lower taxonomic lever of Firmicutes on the right **(E, F, H, I)**. **(D-F)** CKD group; **(G-I)** HC group.

The stacked bar provided an overview of the abundance profiles across samples at phylum, genus, and species levels (**Figures 3A–C**). At the phyla level, Firmicutes, Bacteroidetes and Proteobacteria accounted for more than 98% in each group and were considered as the dominant phyla. Additionally, 49 microbial genera and 54 microbial species exhibiting significant differences between the two groups were identified (P < 0.05). The interactive pie chart outlines the taxonomic compositions for a specific selected group and its lower taxonomic level. As shown in **Figures 3D and G**, the proportion of phylum Firmicutes in the CKD group and the HC group was 53% and 70%, respectively, indicating that the abundance of Firmicutes in patients with CKD was reduced. The CKD group exhibited a significantly greater abundance of Bacteroidetes and Proteobacteria in comparison to the HC group. The results of this study were consistent with previous studies on microbial populations in CKD patients (Lun et al., 2018). A comparison with the HC group shows a noticeable change in the proportion of lower taxonomic level within phylum Firmicutes (**Figures 3E, F, H, I**). For example, we can observe a relative decrease in the abundance of genus *Faecalibacterium* and species *F. prausnitzii* in the CKD group. Firmicutes and Bacteroidetes play an important role in regulating host inflammation and immune function (Chang et al., 2015). The alteration in the ratio of Firmicutes to Bacteroidetes within the microbial community serves as a significant indicator (Mokhtari et al., 2021), reflecting the dysregulation of gut microbiota in patients with CKD.

### Significant bacteria and combinatorial bacterial biomarkers related to CKD

**Figure 4:**
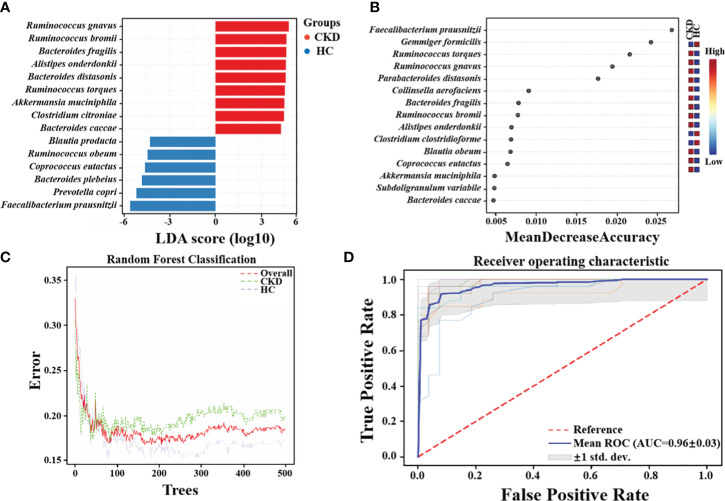
Crucial Bacteria and potential bacterial biomarkers related to CKD. **(A)** The bar graph showing the top 15 significant bacterial taxa of gut microbiome related to CKD (LDA score > 2, *P* < 0.05). **(B)** Features are ranked based on their contributions to classification accuracy (Mean Decrease Accuracy). **(C)**The graph summarizes the classification performance across different groups using Random Forests algorithm. **(D)** The diagnostic potential of gut microbiota for distinguishing CKD and HC are reflected in the ROC curve.

Via LEfSe analysis, nine species, including *Ruminococcus gnavus*, *Ruminococcus bromii*, *Bacteroides fragilis*, *Alistipes onderdonkii*, *Bacteroides distasonis*, *Ruminococcus torques*, *Akkermansia muciniphila*, *Clostridium citroniae*, and *Bacteroides caccae*, showed significantly enriched in CKD (**Figure 4A**). Moreover, six species, namely *Blautia producta*, *Ruminococcus obeum*, *Coprococcus eutactus*, *Bacteroides plebeius*, *Prevotella copri*, and *F. prausnitzii*, exhibited a notable reduction in abundance in CKD patients compared to HC based on linear discriminant analysis (LDA) > 2.0 (all *P* < 0.01). The findings of a study recently conducted by Jiang et al. lend credence to this assumption. Notably, the relative abundance of *F. prausnitzii* was significantly lower in the CKD group compared to the healthy control (HC) group (*P* < 0.01). The random forests model demonstrated high accuracy in predicting disease types based on fecal sample microbiome profiles (**Figure 4C**). The established model exhibited an out-of-bag (OOB) error rate of 18.5%, and a misjudgment rate of 19.8% in predicting the absence of CKD, while the misjudgment rate for predicting CKD was 17.2%. The contributions of different features to the classification accuracy were evaluated and ranked using the mean decrease accuracy metric. Most notably, it was found that *F. prausnitzii* made the greatest contribution in discriminating CKD from HC (**Figure 4B**). The receiver operating characteristic (ROC) was conducted to assess the clinical diagnostic capability of microbial biomarkers. When using microbial features as biomarkers for CKD, the area under the curve (AUC) of the training set is 0.96 (**Figure 4D**).

### Microbial functional prediction of gut microbiota in CKD

**Figure 5:**
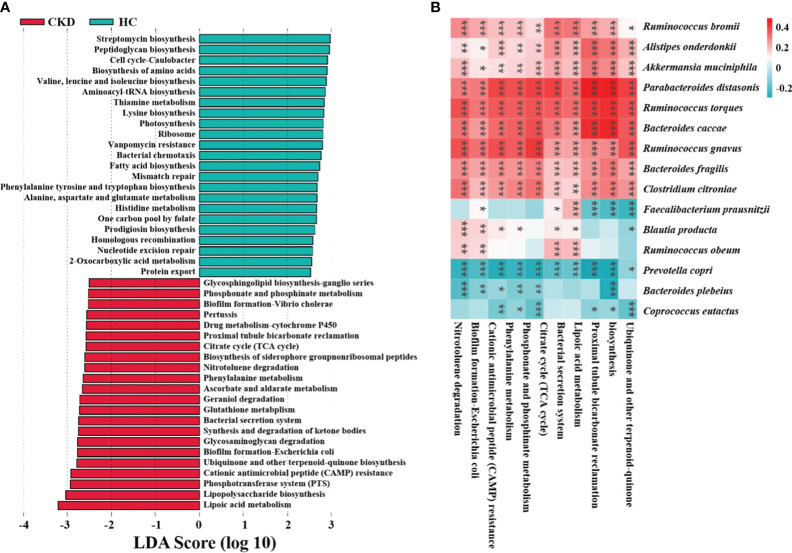
Functional characteristics and regulatory mechanism prediction of gut microbiota in CKD. **(A)** The LEfSe analysis defined characteristic microbial functions based on KEGG pathways for different groups (LDA score > 2.5). **(B)** An example of a spearman correlation between CKD-related microbial species and KEGG pathways appears in the following heatmap. (***, *P* < 0.001; **, *P* < 0.01; *, *P* < 0.05)

To identify how gut microbiota influences CKD, functional prediction was performed using PICRUSt2. In total, 322 pathways were identified, which were divided into six categories (**Supplementary Table A4**). LEfSe analysis revealed the characteristic microbial functions of different groups based on the kyoto encyclopedia of genes and genomes (KEGG) pathway (LDA > 2.5), with 23 related pathways enriched in the HC group and 22 related pathways enriched in the CKD group (**Figure 5A**). Of these, half were associated with inflammation and oxidative stress. Oxidative stress plays an important role in the progression of CKD. The study suggests that burdock fructooligosaccharide can inhibit cell apoptosis and oxidative stress through the Nrf2/HO-1 signaling pathway, protecting renal tubular epithelial cells from damage induced by high glucose (Ding et al., 2021). **Figure 5B** shows the correlation between abundance of CKD-related microbial species and abundance of predicted KEGG pathways. The abundance of *F. prausnitzii* showed a significant association with six major pathways, with negative correlations observed with proximal tubule bicarbonate reclamation, lipoic acid metabolism, and ubiquinone and other terpenoid quinone biosynthesis (*P* < 0.001).

## Discussion

Disturbances in the normal gut microbiome have been associated with the development of various chronic diseases, including cerebrovascular diseases (Liu et al., 2023), obesity (Liu et al., 2017) and diabetic nephropathy (Zhang et al., 2022). Regulating or reshaping gut microbiota is crucial for host health. Li et al. found that polysaccharides and glycosides from Aralia echinocaulis can protect arthritic rats by modulating gut microbiota composition (Li et al., 2021). Wang et al. discovered that Schisandrin improves ulcerative colitis in mice by inhibiting the SGK1/NLRP3 signaling pathway and reshaping gut microbiota (Wang et al., 2023b). We previously reviewed the changes in gut microbiota composition and functionality in CKD patients, explored the inflammatory and immune-related mechanisms by which gut microbiota influence CKD progression, and elucidated the potential application of targeting gut microbiota as a therapeutic approach for CKD (Zhang et al., 2023). There were differences in the composition and diversity of microbial communities in feces from patients with CKD and HC in this study. In patients with CKD, Bacteroidetes and Proteobacteria richness exceeded that of the control individuals dramatically, while Firmicutes richness declined. The main role of Firmicutes is facilitating host energy absorption from the diet (Tabibian et al., 2015), and they are capable of converting polysaccharides into short-chain fatty acids (SCFA) and monosaccharides, which leads to increased energy absorption, and SCFAs can inhibit inflammation and reduce kidney injury (Marzocco et al., 2018). Bacteroidetes can also produce butyric acid, propionic acid, acetic acid, and other SCFAs by fermentation (Hobby et al., 2019). In this study, alterations in the ratio of Firmicutes to Bacteroidetes in the CKD group may indicate the presence of dysbiosis in the gut microbiome and disrupted energy regulation. Moreover, Proteobacteria can induce inflammation by weakening the gut barrier function and synthesizing pro-hypertensive neurotransmitters such as norepinephrine and serotonin. Elevated levels of Proteobacteria may contribute to the development of proteinuric nephropathy and the elevation of blood pressure (Kanbay et al., 2018). Microbes of the genus *Blautia*, *Faecalibacterium*, *Roseburia*, *Coprococcus*, *Lachnospira* and *Bifidobacterium* linked to the generation of SCFAs, particularly butyrate (Louis and Flint, 2009; Jiang et al., 2016). The number of these species was reduced in CKD patients and a reduction in SCFA-producing bacteria is a prominent feature. In this study, fifteen dominant microbial species in CKD and HC groups were identified by LEfSe. Studies have indicated these species such as *Blautia producta* (Yang et al., 2022), *Ruminococcus obeum* (Barathikannan et al., 2019), *Coprococcus eutactus* (Yang et al., 2023), *Bacteroides plebeius* (Pei et al., 2022) and *Prevotella copri* (Jiang et al., 2022), which are found to be in reduced abundance in the CKD group, may have potential effects in alleviating host diseases such as hyperlipidemia and colitis. Among them, as a major producer of butyrate, *F. prausnitzii* had the most significant decrease in relative abundance among CKD patients. As a strict anaerobe, *Faecalibacterium* is a core commensal microbe in the human gut, with an abundance of up to 10%. Among them, *F. prausnitzii* is highly important for the human gut and health. A reduction in this bacterium can lead to a weakening of intestinal anti-inflammatory and immune regulatory capabilities, earning it the title of a “key species” in the gut and being recognized as a next-generation probiotic with therapeutic potential (Lopez-Siles et al., 2017; Khan et al., 2023). Jiang et al. identified a negative correlation between *Roseburia* spp., *F. prausnitzii* and C-reactive protein levels and renal function, suggesting that a decrease in butyrate-producing bacteria may contribute to CKD-associated inflammation and the progression of disease (Jiang et al., 2016). As a result of these findings, the microbial marker-based classifiers are effective in determining whether a patient has CKD or not. Interestingly, it was found that *F. prausnitzii* made the greatest contribution in discriminating CKD from HC. This highlights the potential importance of *F. prausnitzii* for monitoring the progression of CKD. Previous studies have has shown that *F. prausnitzii* exerts its anti-inflammatory effects and enhances renal function through its butyrate metabolite and the renal GPR-43 receptor (Li et al., 2022). Additionally, it has been reported that amylose resistant starch supplementation (HAM-RS2) increased *Faecalibacterium* levels and reduced systemic inflammation in CKD patients (Laffin et al., 2019). Besides, a number of recent studies have reported that the depletion of *F. prausnitzii* in various gastrointestinal disorders, drawing interest in this bacterium as a potential probiotic in the future (Pied et al., 2011; Kabeerdoss et al., 2013; Karlsson et al., 2013; Machiels et al., 2014).

Functionally, the KEGG pathway prediction analysis was categorized into level 1, level 2 and level 3. Level 1 was found to be the most closely associated with metabolism, and level 3 indicated that multiple compounds were being degraded. As a metabolic organ, the kidney can eliminate exogenous substances, including geraniol (Pluym et al., 2022), nitrotoluene (Shen et al., 2023), glycosaminoglycan (Afolayan et al., 2019), and ketone bodies (Halperin and Cheema-Dhadli, 1989). And level 3 related to the biosynthesis of substances, such as ketone bodies, glycosphingolipid (D'Angelo et al., 2013), siderophore group nonribosomal peptides (Swayambhu et al., 2021), lipopolysaccharide (Chen et al., 2015) and ubiquinone and other terpenoid-quinone biosynthesis (Wang et al., 2023a). Remarkably, Wang et al. first reported on the significance of ubiquinone and other terpenoid-quinone biosynthesis pathways in the progression of CKD (Wang et al., 2023a). Chen et al. discovered that microbial genes associated with lipopolysaccharide biosynthesis were more prevalent in advanced stage cases of CKD. Specifically, the serum levels of lipopolysaccharide increased with the severity of renal dysfunction and reached its peak in the advanced stage of CKD (Chen et al., 2015). Additionally, most of the other KEGG pathways are related to metabolism. Phosphate is a key uremic toxin that is linked to unfavorable results. With the progression of CKD, the kidney’s ability to eliminate excessive dietary phosphate diminishes. This reduction in excretion prompts compensatory endocrine responses, leading to the development of CKD-mineral and bone disorder (CKD-MBD) (Favero et al., 2021). Ketteler conducted a review that suggests the need to assess the effectiveness of phosphate-lowering strategies for patients in CKD stages 3-5 in order to slow down the progression of CKD-MBD (Ketteler, 2011). There are several instances of medications metabolized predominantly by cytochrome P450 (CYP) that exhibit modified pharmacokinetics in CKD. The altered CYP metabolism can potentially contribute to variations in drug responses and the occurrence of adverse drug events among CKD patients, necessitating potential dosing adjustments (Ladda and Goralski, 2016). Previous studies have confirmed a negative correlation between phenylalanine levels and diminished renal function, suggesting its potential as a valuable predictor of CKD incidence and prevalence (Lee et al., 2020). Glutathione, a crucial intracellular antioxidant found in nearly all tissues, including the kidney. Dong et al. discovered that rewiring the metabolism of glutathione shields renal tubule cells from apoptosis and ferroptosis induced by cisplatin (Dong et al., 2023).

**Figure 6:**
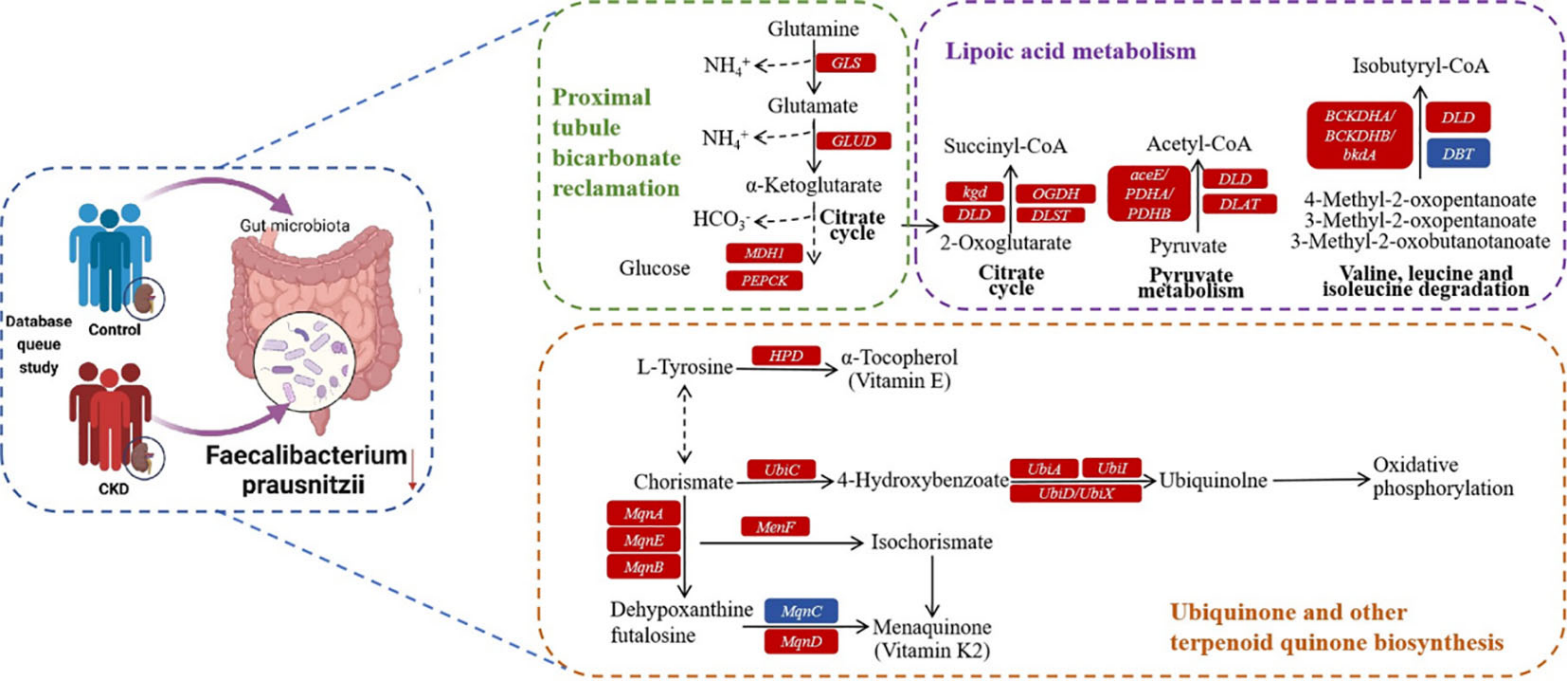
Possible mechanism for the impact of CKD by *F. prausnitzii* summarized in KO genes and KEGG pathways. Representative KO genes are depicted in pathway modules modified from KEGG pathway maps “proximal tubule bicarbonate reclamation”, “lipoic acid metabolism”, and “ubiquinone and other terpenoid-quinone biosynthesis”. Each box represents a KO gene, with red boxes representing elevation or blue boxes representing depletion based on any of the CKD group.

As a commensal bacterium, *F. prausnitzii* possesses anti-inflammatory properties and has been reported to mitigate the extent of acute (Sokol et al., 2008) and chronic (Martín et al., 2014) inflammation in murine models. These beneficial effects are attributed, at least in part, to the secretion of metabolites that can impede NF-κB activation and restrain IL-8 production (Sokol et al., 2008), while simultaneously promoting upregulation of regulatory T cell generation (Qiu et al., 2013). On the basis of the functional prediction analysis, six pathways linked to the abundance of *F. prausnitzii* were identified, and three of these pathways exhibited a negative correlation. Lipoic acid metabolism (Solmonson and DeBerardinis, 2018) and ubiquinone and other terpenoid quinone biosynthesis (Abby et al., 2020) pathways are both closely associated with oxidative stress. Additionally, the proximal tubule bicarbonate reclamation plays a vital role in secreting acid into the renal tubules, facilitating the reabsorption of approximately 80% of bicarbonate (HCO_3_^-^) to regulate the acid-base balance in systemic blood. According to the above findings, a new hypothesis for the mechanism of CKD is presented. *F. prausnitzii* may contribute to regulate acid-base disturbance and pro-oxidant metabolism in the gut and host, thereby ameliorating the progression of CKD. Possible mechanisms for the impact of CKD by *F. prausnitzii* were summarized in KO genes and KEGG pathways (**Figure 6**). Using gut microbiome as a therapeutic target for CKD, this finding is of great significance and might result in the development of new therapies.

Admittedly, one of the main limitations of this study is that we did not differ the clinical staging, the etiology of CKD, and treatment received among the CKD groups, which is due to the uncompleted documentation of the public databases. The disease status, particularly conditions like hypertension (Yan et al., 2020) and diabetes (Mosterd et al., 2021), was the primary factor shaping the microbial profiles associated with CKD. Additionally, differences in treatment methods within the CKD group were not taken into analysis. It has been reported that different hemodialysis techniques can impact the gut microbiome in patients with uremia (He and Xie, 2020). Therefore, it is crucial for future basic and clinical research to assess the influence of these factors on the microbial profile of CKD patients.

## Conclusion

Our study offers a detailed comparison of the variances in the fecal microbiota between CKD and healthy people. Specifically, the influence of *F. prausnitzii* on CKD suggests a crucial connection between metabolic processes and the gut microbiota in the pathogenesis of CKD. Meanwhile, our study identified certain gut bacteria that are potentially associated with CKD as biomarkers. This study offers a novel perspective on how gut microbiota can affect kidney disease in a vast population, and it may benefit future early detection and monitoring of CKD.

## Data availability statement

The original contributions presented in the study are included in the article/**Supplementary Material**. Further inquiries can be directed to the corresponding authors.

## Ethics statement

Ethical approval was not required for the studies involving humans because the data used in this study are 16s rRNA sequencing data from public databases. The studies were conducted in accordance with the local legislation and institutional requirements. Written informed consent for participation was not required from the participants or the participants’ legal guardians/next of kin in accordance with the national legislation and institutional requirements because the data used in this study are 16s rRNA sequencing data from public databases. Written informed consent was not obtained from the individual(s) for the publication of any potentially identifiable images or data included in this article because the data used in this study are 16s rRNA sequencing data from public databases.

## Author contributions

YZ: Writing – review & editing, Writing – original draft, Visualization, Methodology, Investigation, Formal analysis. WCZ: Writing – original draft, Visualization, Investigation, Formal analysis. WL: Writing – review & editing, Methodology, Visualization, Formal analysis. XW: Writing – review & editing, Resources, Methodology, Investigation. GL: Writing – original draft, Methodology, Data curation. JL: Writing – original draft, Project administration, Data curation. JF: Writing – original draft, Visualization, Investigation. XM: Writing – review & editing, Resources, Conceptualization. SJ: Writing – review & editing, Visualization, Investigation. JH: Writing – review & editing, Writing – original draft, Project administration, Methodology, Conceptualization. WJZ: Writing – original draft, Writing – review & editing, Supervision, Project administration, Funding acquisition, Conceptualization. ZZ: Writing – original draft, Writing – review & editing, Validation, Supervision, Funding acquisition, Conceptualization.
