# Supplementary material for: Uncovering specific taxonomic and functional alteration of gut microbiota in chronic kidney disease through 16S rRNA data
Source: Front Cell Infect Microbiol. 2024 Apr 19;14:1363276. doi: 10.3389/fcimb.2024.1363276 (PMC11066246; doi:10.3389/fcimb.2024.1363276)
Supplement: Supplementary file 1 [file Table_1.docx]

**Tables A1 Search Strategy and Search Terms.**

**1.1 PubMed**

| **Sequence** | **Search** | **Hits** |
| --- | --- | --- |
| 1 | (((((((((((((((((((("Renal Insufficiency, Chronic"[Mesh])) OR (Chronic Renal Insufficiencies[Title/Abstract])) OR (Renal Insufficiencies, Chronic[Title/Abstract])) OR (Chronic Renal Insufficiency[Title/Abstract])) OR (Kidney Insufficiency, Chronic[Title/Abstract])) OR (Chronic Kidney Insufficiency[Title/Abstract])) OR (Chronic Kidney Insufficiencies[Title/Abstract])) OR (Kidney Insufficiencies, Chronic[Title/Abstract])) OR (Chronic Kidney Diseases[Title/Abstract])) OR (Chronic Kidney Disease[Title/Abstract])) OR (Disease, Chronic Kidney[Title/Abstract])) OR (Diseases, Chronic Kidney[Title/Abstract])) OR (Kidney Disease, Chronic[Title/Abstract])) OR (Kidney Diseases, Chronic[Title/Abstract])) OR (Chronic Renal Diseases[Title/Abstract])) OR (Chronic Renal Disease[Title/Abstract])) OR (Disease, Chronic Renal[Title/Abstract])) OR (Diseases, Chronic Renal[Title/Abstract])) OR (Renal Disease, Chronic[Title/Abstract])) OR (Renal Diseases, Chronic[Title/Abstract]) | 171,013 |
| 2 | (((((((((((((((( "Microbiota"[Mesh]) OR (Microbiotas[Title/Abstract])) OR (Microbial Community[Title/Abstract])) OR (Community, Microbial[Title/Abstract])) OR (Microbial Communities[Title/Abstract])) OR (Microbial Community Composition[Title/Abstract])) OR (Community Composition, Microbial[Title/Abstract])) OR (Composition, Microbial Community[Title/Abstract])) OR (Microbial Community Compositions[Title/Abstract])) OR (Microbial Community Structure[Title/Abstract])) OR (Community Structure, Microbial[Title/Abstract])) OR (Microbial Community Structures[Title/Abstract])) OR (Microbiome[Title/Abstract])) OR (Microbiomes[Title/Abstract])) OR (Human Microbiome[Title/Abstract])) OR (Human Microbiomes[Title/Abstract])) OR (Microbiome, Human[Title/Abstract]) | 123,972 |
| 3 | 1 AND 2 | 651 |

**1.2 Web of science**

| **Sequence** | **Search** | **Hits** |
| --- | --- | --- |
| 1 | TS=(Renal Insufficiency, Chronic) OR AB=(Chronic Renal Insufficiencies OR Renal Insufficiencies, Chronic OR Chronic Renal Insufficiency OR Kidney Insufficiency, Chronic OR Chronic Kidney Insufficiency OR Chronic Kidney Insufficiencies OR Kidney Insufficiencies, Chronic OR Chronic Kidney Diseases OR Chronic Kidney Disease OR Disease, Chronic Kidney OR Diseases, Chronic Kidney OR Kidney Disease, Chronic OR Kidney Diseases, Chronic OR Chronic Renal Diseases OR Chronic Renal Disease OR Disease, Chronic Renal OR Diseases, Chronic Renal OR Renal Disease, Chronic OR Renal Diseases, Chronic) | [167,1](https://www.webofscience.com/wos/woscc/summary/201acb92-3dc3-47e2-abfe-d3bee307b0e9-912f62c8/relevance/1)86 |
| 2 | TS=(Microbiota) OR AB=(Microbiotas OR Microbial Community OR Community, Microbial OR Microbial Communities OR Microbial Community Composition OR Community Composition, Microbial OR Composition, Microbial Community OR Microbial Community Compositions OR Microbial Community Structure OR Community Structure, Microbial OR Microbial Community Structures OR Microbiome OR Microbiomes OR Human Microbiome OR Human Microbiomes OR Microbiome, Human) | [254,76](https://www.webofscience.com/wos/woscc/summary/a3c674c6-ee6e-49c1-b72c-eb0c7a3af634-912f7206/relevance/1)9 |
| 3 | 1 AND 2 | 1161 |

**1.3 GM repo**

| **Sequence** | **Search** | **Hits** |
| --- | --- | --- |
| 1 | the phenotype was “kidney failure, chronic” | 1 |
| 2 | it has healthy controls |  |
| 3 | the percentage of failed runs was set to less than 90% |  |
