# Supplementary material for: Uncovering specific taxonomic and functional alteration of gut microbiota in chronic kidney disease through 16S rRNA data
Source: Front Cell Infect Microbiol. 2024 Apr 19;14:1363276. doi: 10.3389/fcimb.2024.1363276 (PMC11066246; doi:10.3389/fcimb.2024.1363276)
Supplement: Supplementary file 2 [file Table_2.docx]

**Table A2 Inclusion and exclusion criteria of this study.**

| Selection criteria | Content |
| --- | --- |
| Inclusion criteria | Participants include individuals with CKD and healthy individuals. |
|  | Characteristic changes in gut microbiota were reported. |
|  | Clinical studies including cross-sectional studies, cohort studies, and case-control studies. |
| Exclusion criteria | Literature that is duplicated and cannot be accessed in full text |
|  | Gut microbiota sequencing data obtained from metagenomics sequencing or RT-PCR or public databases |
|  | Inability to access original 16S rRNA gene high-throughput sequencing data or sequencing data quality not meeting the standards. |
