# Supplementary material for: Uncovering specific taxonomic and functional alteration of gut microbiota in chronic kidney disease through 16S rRNA data
Source: Front Cell Infect Microbiol. 2024 Apr 19;14:1363276. doi: 10.3389/fcimb.2024.1363276 (PMC11066246; doi:10.3389/fcimb.2024.1363276)
Supplement: Supplementary file 3 [file Table_3.docx]

**Table A3 Characteristics of included projects in the quantitative synthesis.**

| **Study** | **Numbers in Study** | **Gender (female/male)** | | | **Age (years)** | | | **BMI (kg/m2)** | | | **eGFR (mL/min/1.73m^2^)** | | |
| --- | --- | --- | --- | --- | --- | --- | --- | --- | --- | --- | --- | --- | --- |
|  |  | **CKD** | **HC** | ***p*** | **CKD** | **HC** | ***p*** | **CKD** | **HC** | ***p*** | **CKD** | **HC** | ***p*** |
| S1 | n = 200 | 50/50 | 50/50 | 1.00 | 56.64 ± 17.25 | 60.64 ± 16.51 | 0.101 | 25.08 ± 3.59 | 23.62 ± 2.75 | 0.002 | 56.95 ± 39.27 | 100.90 ± 9.41 | <0.001 |
| S2 | Discovery (n = 320) | 50/60 | 105/105 | 0.44 | 51.75 ± 14.60 | 50.02 ± 4.56 | 0.229 | 13.35 ± 1.37 | 13.47 ± 2.22 | 0.547 | 37.18 ± 35.94 | 104.29 ± 9.78 | <0.001 |
|  | Validation (n = 112) | 20/29 | 30/33 | 0.472 | 50.20 ± 16.09 | 46.24 ± 7.66 | 0.116 | 23.13 ± 1.68 | 23.18 ± 2.13 | 0.894 | 57.83 ± 63.85 | 103.38 ± 10.75 | <0.001 |
| S3 | n = 30 | HD: 5/10 | 12/9 | NS | HD: 61 (54; 71) | 58 (53/62) | NS | 23,7 (21,5; 30,8) | 25,3 (23,4;25,8) | NS | HD: 6.0 (5.9; 9.3) | 77.6 (73.4; 86.6) | <0.001 |
|  |  | PD: 3/12 |  | NS | PD: 62 (54; 69) |  | NS | 28,7 (23,9; 32,6) |  | NS | PD: 7.9 (7.3; 14.0) |  | <0.001 |
| S4 | n = 79 | 21/43 | 11/4 | 0.007 | 80.7 ± 6.2 | 73.7 ± 7.6 | 0.0003 | 28.4 ± 4.7 | 25.5 ± 2.9 | 0.0301 | 26 ± 11 | 75 ± 11 | <0.0001 |
| S5 | n = 105 | CKD: 14/18 | 14/16 | NA | CKD: 46.12 ± 11.39 | 44.71 ± 10.21 | 0.76 | 23.07 ± 2.77 | 23.56 ± 3.37 | 0.97 | CKD: 47.75 ± 18.72 | 101.42 ± 10.99 | <0.0001 |
|  |  | CKD-HTN: 22/21 |  | NA | CKD-HTN: 49.76 ± 13.23 |  | 0.43 | 24.02 ± 3.06 |  | 0.43 | CKD-HTN: 11.78 ± 5.64 |  | <0.0001 |
| S6 | n = 20 | 5/5 | 4/6 | NS | 59 ± 16 | 54.8 ± 10.4 | NS | 22.8 ± 3.8 | 22.3 ± 3.6 | NS | 15.8 ± 11.5 | NA | NA |

CKD, chronic kidney disease; HC, healthy controls; HD, haemodialysis; PD, peritoneal dialysis; CKD-HTN, CKD with hypertension; Discovery, discovery cohort; Validation, validation cohort; BMI, body mass index; eGFR, estimated glomerular filtration rate; NA, not applicable; NS, no statistical significance; *p, p-*values (< 0.05 were considered statistically signifcant).
